# Improving Implantation Rate in 2nd ICSI Cycle through Ovarian Stimulation with FSH and LH in GNRH Antagonist Regimen

**DOI:** 10.1055/s-0041-1736306

**Published:** 2021-11-16

**Authors:** Amanda Souza Setti, Daniela Paes de Almeida Ferreira Braga, Assumpto Iaconelli, Edson Borges

**Affiliations:** 1 Fertility Medical Group, São Paulo, SP, Brazil

**Keywords:** follicle-stimulating hormone, intracytoplasmic sperm injection implantation, luteinizing hormone, ovarian stimulation, hormônio folículo- estimulante, microinjeção intracitoplasmática de espermatozoides, hormônio luteinizante, estímulo ovariano

## Abstract

**Objective** To investigate whether patients with a previous recombinant follicle stimulating hormone (rFSH)-stimulated cycle would have improved outcomes with rFSH + recombinant luteinizing hormone (rLH) stimulation in the following cycle.

**Methods** For the present retrospective case-control study, 228 cycles performed in 114 patients undergoing intracytoplasmic sperm injection (ICSI) between 2015 and 2018 in an in vitro fertilization (IVF) center were evaluated. Controlled ovarian stimulation (COS) was achieved with rFSH (Gonal-f, Serono, Geneva, Switzerland) in the first ICSI cycle (rFSH group), and with rFSH and rLH (Pergoveris, Merck Serono S.p.A, Bari, Italy) in the second cycle (rFSH + rLH group). The ICSI outcomes were compared among the groups.

**Results** Higher estradiol levels, oocyte yield, day-3 high-quality embryos rate and implantation rate, and a lower miscarriage rate were observed in the rFSH + rLH group compared with the rFSH group. In patients < 35 years old, the implantation rate was higher in the rFSH + rLH group compared with the rFSH group. In patients ≥ 35 years old, higher estradiol levels, oocyte yield, day-3 high-quality embryos rate, and implantation rate were observed in the rFSH + rLH group. In patients with ≤ 4 retrieved oocytes, oocyte yield, mature oocytes rate, normal cleavage speed, implantation rate, and miscarriage rate were improved in the rFSH + rLH group. In patients with ≥ 5 retrieved oocytes, higher estradiol levels, oocyte yield, and implantation rate were observed in the rFSH + rLH group.

**Conclusion** Ovarian stimulation with luteinizing hormone (LH) supplementation results in higher implantation rates, independent of maternal age and response to COS when compared with previous cycles stimulated with rFSH only. Improvements were also observed for ICSI outcomes and miscarriage after stratification by age and retrieved oocytes.

## Resumo

**Objetivo** : Investigar se há algum efeito da suplementação com hormônio luteinizante (LH, na sigla em inglês) no regime com antagonista do hormônio liberador de gonadotropina (GnRH, na sigla em inglês) sobre os resultados dos ciclos consecutivos de injeção intracitoplasmática de espermatozoides (ICSI, na sigla em inglês).

**Métodos** Para o presente estudo retrospectivo de caso-controle, foram avaliados 228 ciclos de microinjeção intracitoplasmática de espermatozoides (ICSI, na sigla em inglês) realizados em 114 pacientes entre 2015 e 2018 em um centro privado de fertilização in vitro (FIV) afiliado a uma universidade. O estímulo ovariano controlado (EOC) foi feito com hormônio folículo- estimulante recombinante (rFSH, na sigla em inglês) (Gonal-f, Serono, Genebra, Suíça) no primeiro ciclo de ICSI (grupo rFSH), e com rFSH e rLH (Pergoveris, Merck Serono S.p.A, Bari, Itália) no segundo ciclo (grupo rFSH + rLH). Os desfechos dos ciclos de ICSI foram comparados entre os grupos.

**Resultados** Níveis mais elevados de estradiol, de recuperação oocitária, taxa de embriões de alta qualidade no 3° dia e taxa de implantação, e menor taxa de aborto foram observados no grupo rFSH + rLH. Em pacientes < 35 anos, a taxa de implantação foi maior no grupo rFSH + rLH em comparação com o grupo rFSH. Em pacientes com ≥ 35 anos, maiores níveis de estradiol, recuperação oocitária, a taxa de embriões de alta qualidade no 3° dia e a taxa de implantação foram observados no grupo rFSH + rLH. Em pacientes com baixa resposta ao EOC (≤ 4 oócitos recuperados), a recuperação oocitária, a taxa de oócitos maduros, a taxa de velocidade normal de clivagem, a taxa de implantação e a taxa de aborto foram melhoradas no grupo rFSH + rLH. Em pacientes com resposta normal ao EOC (≥ 5 oócitos recuperados), níveis mais elevados de estradiol, recuperação oocitária e taxa de implantação foram observados no grupo rFSH + rLH.

**Conclusão** A estimulação ovariana com suplementação de LH resultou em taxas de implantação mais altas, independentemente da idade materna e da resposta ao EOC, em comparação com os ciclos anteriores estimulados apenas com rFSH. Melhorias também foram observadas nos resultados da ICSI e na taxa de aborto quando as pacientes foram estratificadas por idade e número de oócitos recuperados.

## Introduction

For assisted reproductive technology (ART), gonadotropin-releasing hormone (GnRH) and gonadotropins are routinely administered for controlled ovarian stimulation (COS). For that, recombinant follicle stimulating hormone (rFSH or follitropin alfa) and recombinant luteinizing hormone (rLH or lutropin alfa) are the key hormonal stimulus, which can be used individually or in combination. Follicle stimulating hormone and LH play distinct but complementary roles in follicle regulation, leading to synergistic actions in stimulating the recruitment and development of ovarian follicles, increasing follicle estradiol secretion, and completing oocyte maturation and subsequent ovulation. 1

Although ovarian stimulation is essential for the success of ART, it is also known to reduce endogenous FSH and LH releases. 2 Particularly, GnRH antagonists induce a profound pituitary supression, avoiding premature LH surge. Consequently, recruited follicles are radically deprived of LH sustenance. Exogenous FSH stimulation will support follicular development in most patients undergoing ART; however, up to 12% of the patients will not respond to FSH stimulation alone, which can happen due to the absence of LH. 3 For this subpopulation of patients, there is evidence that LH supplementation to FSH administration could be advantageous. 3 4 5 6 7 8 9 10

In fact, rLH was originally commercialized to supplement follitropin alfa administration for specific patients, especially those presenting with severe LH and FSH deficiency, namely hypogonadotropic hypogonadism. More recently, new products were developed in a fixed combination of 2:1 (150 IU of rFSH and 75 IU of rLH), under the presupposition that this is the optimal FSH:LH ratio for the purpose of stimulating follicular development. Then, it was agreed that patients (i) with previous poor response to ovarian stimulation, (ii) inadequate ovarian response in the treatment in progress, (iii) aged ≥ 35 years old could also benefit from LH supplementation. 10

A recent meta-analysis that included 36 randomized controlled trials investigated the effectiveness of rLH combined with rFSH for COS compared with rFSH alone in 8,125 women undergoing ART. Moderate quality evidence that the use of rLH combined with rFSH may lead to more ongoing pregnancies than rFSH alone was observed. No evidence of a difference between the two regimens was observed in terms of live birth rate. The authors concluded that the evidence was insufficient to encourage or discourage stimulation regimens that include rLH combined with rFSH in ART. 11

To date, there is no evidence that ovarian simulation with rLH improves ART outcomes in an unselected subpopulation. In addition, there is only one previous study that investigated cycles in which patients acted as their own controls. The objective of the present study was to investigate whether patients with a previous rFSH-stimulated cycle would have improved outcomes with rFSH + rLH stimulation in the following cycle.

## Methods

### Experimental Design, Patients, and Inclusion and Exclusion Criteria

The present case-control within-subject study included data obtained via chart review of 228 cycles performed in 114 patients undergoing ICSI between 2015 and 2018 in a private university-affiliated IVF center. For all patients, rFSH (Gonal-f, Serono, Geneva, Switzerland) was used for COS in the first ICSI cycle (rFSH group, *n* = 114), followed by ovarian stimulation with rFSH and rLH (Pergoveris, Merck Serono S.p.A, Bari, Italy) in the next cycle (rFSH + rLH group, *n* = 114). Pituitary suppression was achieved with GnRH antagonist (cetrorelix acetate, Cetrotide; Merck KGaA, Darmstadt, Germany) in both groups.

The inclusion criteria were: couples with primary infertility undergoing their first rFSH-stimulated ICSI cycle, with intended fresh embryo transfer on day 5 of embryo development, who underwent a second rFSH + rLH stimulated ICSI cycle, also intending fresh embryo transfer on day 5 of embryo development.

The exclusion criteria were as follows: Female patients undergoing ICSI cycles with vitrified/thawed or donated oocytes, surgical sperm retrieval, cryopreserved sperm, and vitrified/thawed embryo transfer.

Ovarian response to COS and ICSI outcomes were compared between the groups.

All patients signed a written informed consent form. The present study was approved by the local Institutional Review Board.

### Controlled Ovarian Stimulation

For the first ICSI cycle of the patients, COS was started on the 3 ^rd^ day of the cycle, with the administration of daily doses of r-FSH. For the second ICSI cycle, on the 3 ^rd^ day of the cycle, COS was started with the administration of r-FSH + r-LH.

The following steps were the same for both the first and the second ICSI cycles. When at ≥ 1 follicle ≥ 14 mm was visualized, pituitary blockage was performed using GnRHa. When ≥ 3 follicles attained a mean diameter ≥ 17 mm and adequate serum estradiol levels were observed, final follicular maturation was triggered by the administration of 250 µg of r-hCG (Ovidrel, Merck KGaA, Geneva, Switzerland) or GnRH agonist (triptorelin 0.2 mg, Gonapeptyl; Ferring GmbH, Kiel, Germany; or leuprolide acetate 2.0mg, Lupron Kit, Abbott S.A Societé Française des Laboratoires, Paris, France). Oocyte retrieval was performed 35 hours later.

### Intracytoplasmic Sperm Injection and Embryo Quality and Transfer

Intracytoplasmic sperm injection was performed according to Palermo et al. 12 Embryos were cultured in 50-µL drops culture medium (Global, LifeGlobal, Guilford, USA) covered with paraffin oil, in a humidified atmosphere under 6% CO _2,_ at 37°C, for 5 days. The embryos were morphologically evaluated on days 3 and 5 of development. On day 5, 1 to 2 embryos were transferred per patient, depending on maternal age and embryo quality, using a soft catheter with transabdominal ultrasound guidance.

### Clinical Follow-Up

A serum pregnancy test was performed 10 days after embryo transfer. Women with a positive β human Chorionic Gonadotropin (βhCG) test underwent a transvaginal ultrasound scan after 2 weeks. Clinical pregnancy was confirmed when at least one intrauterine gestational sac with fetal heartbeat was detected. Implantation rate was calculated per transferred embryos. Clinical pregnancy rates were calculated per embryo transfer. Miscarriage was defined as pregnancy loss before 20 weeks of gestation.

### Data Analysis and Statistics

The sample size calculation suggested that 200 cycles would be enough to demonstrate a 20% effect with 80% power and 5% significance level considering as primary outcome the implantation rate.

In the first analysis, response to COS, and the outcomes of ICSI were compared between the rFSH and rFSH + rLH groups ( *n* = 228), using generalized linear models followed by the Bonferroni post hoc test. Then, data were stratified according to female age (< 35 years old, *n* = 50, and ≥ 35 years old, *n* = 178) and response to COS (poor response: ≤ 4 retrieved oocytes, *n* = 102, and normal response: ≥ 5 retrieved oocytes, *n* = 126), and were reanalyzed as mentioned above. In all models, female age, body mass index (BMI) and total FSH dose were included as covariates. No patient has shifted age categories from the 1 ^st^ to the 2 ^nd^ ICSI cycle. Patients that became pregnant in the 1 ^st^ ICSI cycle and returned for a 2 ^nd^ cycle desiring another child were not excluded from the analysis, to avoid bias.

Data are expressed as mean ± standard error for continuous variables or as percentages for dichotomous variables, and p-values. P-value was significant at 5% level (< 0.05). The analysis was performed using IBM SPSS Statistics for Windows, version 21 (IBM Corp., Armonk, NY, USA).

## Results

All patients completed the follow-up (20 weeks of gestation), and there were no data missing regarding the reported variables. Higher estradiol levels (1151.73 ± 194.34 pg/mL versus 1909.11 ± 194.34 pg/mL, *p* = 0.006), oocyte yield (63.41 versus 69.78%, *p* = 0.045), day-3 high-quality embryos rate (34.13 versus 47.71%, *p* = 0.029) and implantation rate (18.57 versus 26.47%, *p* < 0.001), and lower miscarriage rate (33.0 versus 5.0, *p* = 0.031) were observed in the rFSH + rLH group compared with the rFSH group ( Table 1 ).

**Table 1:** Descriptive analysis of demographics, response to COS and laboratorial ICSI outcomes of patients in repeated cycles ( *n* = 228)

Variables	rFSH group ( *n* = 114)	rFSH + rLH group ( *n* = 114)	*p-value*
Female age	37.19 ± 0.35	37.89 ± 0.35	0.160
Male age	39.23 ± 0.63	39.96 ± 0.64	0.416
BMI	24.88 ± 0.42	24.68 ± 0.42	0.740
FSH dose (IU)	2826.92 ± 199.67	2693.64 ± 198.79	0.636
LH dose (IU)	0.0	1346.82 ± 34.50	NA
Estradiol level (pg/mL)	1151.73 ± 194.34	1909.11 ± 194.34	0.006
Cycles triggered with GnRHa	9/114 (7.9)	10/114 (8.8)	0.811
Follicles (n)	9.99 ± 0.70	10.38 ± 0.70	0.695
Retrieved oocytes (n)	6.37 ± 0.49	7.30 ± 0.49	0.185
Oocyte yield (%)	63.41 ± 2.24	69.78 ± 2.24	0.045
MII oocyte rate (%)	67.72 ± 2.53	71.48 ± 2.52	0.293
Fertilization rate (%)	77.33 ± 2.41	73.02 ± 2.37	0.202
Normal cleavage speed rate (%)	67.16 ± 3.16	73.07 ± 3.11	0.182
D3 high –quality embryos rate (%)	34.13 ± 4.37	47.71 ± 4.40	0.029
Blastocyst development rate (%)	36.72 ± 6.68	42.68 ± 5.73	0.499
Frozen embryos (n)	2.21 ± 0.61	3.05 ± 0.57	0.308
Endometrial thickness (mm)	10.32 ± 0.27	10.71 ± 0.25	0.288
Embryos transferred (n)	2.08 ± 0.09	2.04 ± 0.09	0.759
Cycles with embryo transfer (%)	70/114 (61.4)	69/114 (60.5)	0.892
Implantation rate (%)	18.57 ± 0.52	26.47 ± 0.62	< 0.001
Pregnancy rate (%)	15/70 (21.4)	20/69 (29.0)	0.303
Miscarriage rate (%)	5/15 (33.0)	1/20 (5.0)	0.031
OHSS rate (%)	3/114 (2.6)	6/114 (5.3)	0.308

Abbreviations: BMI, body mass index; COS, controlled ovarian stimulation; D3, day 3 of embryo development; ICSI, intracytoplasmic sperm injection; IU, international unit; MII, metaphase II; NA, not applicable; OHSS, ovarian hyper stimulation syndrome; rFSH, recombinant follicle stimulating hormone; rLH, recombinant luteinizing hormone.

Note: values are mean ± standard error, unless otherwise noted.

In patients < 35 years old, the implantation rate was significantly higher in the rFSH + rLH group compared with the rFSH group (21.43 versus 38.46%, *p* < 0.001) ( Table 2 ).

**Table 2:** Descriptive analysis of demographics, response to COS and laboratorial ICSI outcomes of patients < 35 years old in repeated cycles ( *n* = 50)

Variable	rFSH group ( *n* = 25)	rFSH + rLH group ( *n* = 25)	*p-value*
Female age	32.00 ± 0.48	32.05 ± 0.54	0.942
Male age	36.26 ± 1.39	36.31 ± 1.66	0.981
BMI	25.54 ± 0.98	24.58 ± 1.03	0.500
FSH dose (IU)	2521.50 ± 138.76	2471.05 ± 159.17	0.811
LH dose (IU)	0.0	1235.53 ± 401.07	NA
Estradiol level (pg/mL)	1085.05 ± 399.49	1916.20 ± 357.31	0.322
Cycles triggered with GnRHa	1/25 (4.0)	2/25 (8.0)	0.551
Follicles (n)	12.12 ± 1.49	13.74 ± 1.71	0.475
Retrieved oocytes (n)	8.12 ± 1.09	9.84 ± 1.25	0.298
Oocyte yield (%)	70.98 ± 4.49	75.54 ± 5.16	0.505
MII oocyte rate (%)	68.08 ± 4.75	67.36 ± 5.44	0.920
Fertilization rate (%)	81.08 ± 4.47	72.83 ± 5.2	0.229
Normal cleavage speed rate (%)	71.79 ± 5.13	75.32 ± 5.97	0.654
D3 high-quality embryos rate (%)	42.97 ± 9.99	40.41 ± 12.24	0.871
Blastocyst development rate (%)	41.60 ± 12.12	47.44 ± 11.06	0.859
Frozen embryos (n)	1.50 ± 0.56	2.83 ± 0.64	0.118
Endometrial thickness (mm)	10.30 ± 0.59	10.87 ± 0.66	0.520
Embryos transferred (n)	2.33 ± 0.14	2.07 ± 0.17	0.223
Cycles with embryo transfer (%)	21/25 (84.0)	18/25 (72.00)	0.409
Implantation rate (%)	21.43 ± 1.01	38.46 ± 1.72	< 0.001
Pregnancy rate (%)	6/21 (28.57)	9/18 (50.00)	0.197
Miscarriage rate (%)	2/6 (33.33)	0/9 (0.0)	0.083
OHSS rate (%)	0/25 (0.0)	1/25 (4.0)	0.999

Abbreviations: BMI, body mass index; COS, controlled ovarian stimulation; D3, day 3 of embryo development; ICSI, intracytoplasmic sperm injection; IU, international unit; MII, metaphase II; NA, not applicable; OHSS, ovarian hyper stimulation syndrome; rFSH, recombinant follicle stimulating hormone; rLH, recombinant luteinizing hormone.

Note: values are mean ± standard error, unless otherwise noted.

In patients aged ≥ 35 years old, higher estradiol levels (1161.80 ± 215.94 pg/mL versus 1966.55 ± 220.13 pg/mL, *p* = 0.009), oocyte yield (61.28%versus 68.62%, *p* = 0.038), day-3 high-quality embryos rate (32.01 versus 48.81%, *p* = 0.013), and implantation rate (17.35 versus 23.64%, *p* < 0.001) were observed in the rFSH + rLH group compared with the rFSH group ( Table 3 ).

**Table 3:** Descriptive analysis of demographics, response to COS and laboratorial ICSI outcomes of patients aged ≥ 35 years old in repeated cycles in patients ( *n* = 178)

Variable	rFSH group ( *n* = 89)	rFSH + rLH group ( *n* = 89)	p-value
Female age	38.65 ± 0.29	39.06 ± 0.28	0.303
Male age	40.10 ± 0.68	40.67 ± 0.66	0.549
BMI	24.70 ± 0.47	24.70 ± 0.45	0.995
FSH dose (IU)	2913.69 ± 248.86	2738.16 ± 2369.51	0.611
LH dose (IU)	0.0	1369.08 ± 359.66	NA
Estradiol level (pg/mL)	1161.80 ± 215.94	1966.55 ± 220.13	0.009
Cycles triggered with GnRHa	8/89 (9.0)	8/89 (9.0)	> 0.999
Follicles (n)	9.39 ± 0.77	9.71 ± 0.75	0.772
Retrieved oocytes (n)	5.88 ± 0.54	6.79 ± 0.53	0.227
Oocyte yield (%)	61.28 ± 2.54	68.62 ± 2.46	0.038
MII oocyte rate (%)	67.61 ± 2.95	72.33 ± 2.83	0.248
Fertilization rate (%)	76.20 ± 2.81	73.06 ± 2.65	0.417
Normal cleavage speed rate (%)	65.72 ± 3.78	72.65 ± 3.57	0.184
D3 high-quality embryos rate (%)	32.01 ± 4.83	48.81 ± 4.69	0.013
Blastocyst development rate (%)	39.06 ± 7.34	45.10 ± 6.24	0.531
Frozen embryos (n)	2.42 ± 0.75	3.09 ± 0.67	0.508
Endometrial thickness (mm)	10.33 ± 0.30	10.68 ± 0.26	0.386
Embryos transferred (n)	1.98 ± 0.11	2.04 ± 0.10	0.712
Cycles with embryo transfer (%)	49/89 (55.06)	51/89 (57.30)	0.698
Implantation rate (%)	17.35 ± 0.60	23.64 ± 0.66	< 0.001
Pregnancy rate (%)	9/49 (18.37)	12/51 (23.53)	0.508
Miscarriage rate (%)	3/9 (33.33)	1/12 (8.33)	0.140
OHSS rate (%)	3/89 (3.4)	5/89 (5.6)	0.469

Abbreviations: BMI, body mass index; COS, controlled ovarian stimulation; D3, day 3 of embryo development; ICSI, intracytoplasmic sperm injection; IU, international unit; MII, metaphase II; NA, not applicable; OHSS, ovarian hyper stimulation syndrome; rFSH, recombinant follicle stimulating hormone; rLH, recombinant luteinizing hormone.

Note: values are mean ± standard error, unless otherwise noted.

In patients with poor response to COS (≤ 4 retrieved oocytes), oocyte yield (56.82 versus 63.29%, *p* = 0.001), mature oocytes rate (69.87 versus 78 + 12%, *p* < 0.001), normal cleavage speed (62.5 versus 75.83%, *p* < 0.001), implantation rate (10.00 versus 20.45%, *p* < 0.001) and miscarriage rate (100 versus 0.00%, *p* < 0.001) were improved in the rFSH + rLH group compared with the rFSH group ( Table 4 ).

**Table 4:** Descriptive analysis of demographics, response to COS and laboratorial ICSI outcomes in repeated cycles in patients with poor response to COS (≤ 4 retrieved oocytes) ( *n* = 102)

Variable	rFSH group ( *n* = 51)	rFSH + rLH group ( *n* = 51)	*p-value*
Female age	38.37 ± 0.54	38.93 ± 0.58	0.481
Male age	39.90 ± 0.89	39.97 ± 0.98	0.959
BMI	25.11 ± 0.45	24.23 ± 0.50	0.194
FSH dose (IU)	3051.60 ± 456.34	2536.05 ± 492.09	0.442
LH dose (IU)	0.0	1268.02 ± 442.97	NA
Estradiol level (pg/mL)	596.24 ± 101.87	725.51 ± 111.24	0.391
Cycles triggered with GnRHa	0/51 (0.0)	0/51 (0.0)	> 0.999
Follicles (n)	4.65 ± 0.30	4.33 ± 0.33	0.472
Retrieved oocytes (n)	2.29 ± 0.16	2.47 ± 0.18	0.481
Oocyte yield (%)	56.82 ± 1.31	63.29 ± 1.34	0.001
MII oocyte rate (%)	69.87 ± 1.34	78 + 12 ± 1.56	< 0.001
Fertilization rate (%)	79.46 ± 1.68	81.0 ± 1.8	0.533
Normal cleavage speed rate (%)	62.5 ± 1.08	75.83	< 0.001
D3 high-quality embryos rate (%)	32.47 ± 6.44	49.14 ± 7.32	0.087
Blastocyst development rate (%)	32.81 ± 7.23	33.20 ± 6.41	0.967
Frozen embryos (n)	0.71 ± 0.17	0.69 ± 0.19	0.957
Endometrial thickness (mm)	10.22 ± 0.45	10.10 ± 0.50	0.861
Embryos transferred (n)	1.68 ± 0.12	1.68 ± 0.13	0.992
Cycles with embryo transfer (%)	25/51 (49.02)	26/51 (50.98)	0.836
Implantation rate (%)	10.00 ± 0.63	20.45 ± 0.96	< 0.001
Pregnancy rate (%)	3/25 (12.00)	6/26 (23.08)	0.332
Miscarriage rate (%)	3/3 (100)	0/6 (0.0)	< 0.001
OHSS rate (%)	0/51 (0.0)	0/51 (0.0)	0.999

Abbreviations: BMI, body mass index; COS, controlled ovarian stimulation; D3, day 3 of embryo development; ICSI, intracytoplasmic sperm injection; IU, international unit; MII, metaphase II; NA, not applicable; OHSS, ovarian hyper stimulation syndrome; rFSH, recombinant follicle stimulating hormone; rLH, recombinant luteinizing hormone.

Note: values are mean ± standard error, unless otherwise noted.

In patients with normal response to COS (≥ 5 retrieved oocytes), higher estradiol levels (1725.74 ± 303.65 pg/mL versus 2788.37 ± 281.12 pg/mL, *p* = 0.010), oocyte yield (75.37 versus 82.69%, *p* = 0.006), and implantation rate (23.33 versus 29.35%, *p* < 0.001) were observed in the rFSH + rLH group compared with the rFSH group ( Table 5 ).

**Table 5:** Descriptive analysis of demographics, response to COS and laboratorial ICSI outcomes in repeated cycles in patients with normal response to COS (≥ 5 retrieved oocytes) ( *n* = 126)

Variable	rFSH group ( *n* = 63)	rFSH + rLH group ( *n* = 63)	*p-value*
Female age	36.24 ± 0.44	37.27 ± 0.42	0.092
Male age	38.78 ± 0.86	39.95 ± 0.83	0.322
BMI	24.68 ± 0.66	24.94 ± 0.60	0.770
FSH dose (IU)	2648.61 ± 75.42	2789.08 ± 71.04	0.175
LH dose (IU)	0.0	1394.54 ± 308.62	NA
Estradiol level (pg/mL)	1725.74 ± 303.65	2788.37 ± 281.12	0.010
Cycles triggered with GnRHa	9/63 (14.3)	10/63 (15.9)	0.803
Follicles (n)	14.32 ± 0.91	14.04 ± 0.86	0.826
Retrieved oocytes (n)	9.67 ± 0.61	10.23 ± 0.57	0.503
Oocyte yield (%)	75.37 ± 1.99	82.69 ± 1.78	0.006
MII oocyte rate (%)	73.33 ± 1.43	71.8 ± 1.69	0.489
Fertilization rate (%)	78.31 ± 2.65	72.40 ± 2.51	0.105
Normal cleavage speed rate (%)	70.02 ± 3.16	72.54 ± 3.00	0.565
D3 high-quality embryos rate (%)	35.80 ± 5.96	46.78 ± 5.45	0.174
Blastocyst development rate (%)	41.79 ± 11.63	53.29 ± 9.59	0.445
Frozen embryos (n)	3.71 ± 0.99	4.23 ± 0.80	0.680
Endometrial thickness (mm)	10.40 ± 0.33	10.98 ± 0.27	0.182
Embryos transferred (n)	2.29 ± 0.107	2.21 ± 108	0.603
Cycles with embryo transfer (%)	45/63 (71.43)	42/63 (66.67)	0.513
Implantation rate (%)	23.33 ± 0.72	29.35 ± 0.80	< 0.001
Pregnancy rate (%)	12/43 (27.91)	13/42 (30.95)	0.579
Miscarriage rate (%)	2/12 (16.67)	1/13 (7.69)	0.425
OHSS rate (%)	3/63 (4.76)	6/63 (9.52)	0.299

Abbreviations: BMI, body mass index; COS, controlled ovarian stimulation; D3, day 3 of embryo development; ICSI, intracytoplasmic sperm injection; IU, international unit; MII, metaphase II; NA, not applicable; OHSS, ovarian hyper stimulation syndrome; rFSH, recombinant follicle stimulating hormone; rLH, recombinant luteinizing hormone.

Note: values are mean ± standard error, unless otherwise noted.

## Discussion

In the present study, we observed that COS with rFSH + rLH resulted in higher estradiol levels, oocyte yield, day-3 high-quality embryos rate and implantation rate, and lower miscarriage rate compared with COS with rFSH only. The only previous study that has investigated the effect of adding rLH to stimulation in patients with a previous cycle stimulated with rFSH alone showed lower fertilization rates associated with rLH supplementation. 13

The use of rLH during COS is a matter of debate in the literature that has produced controversial results. In studies that investigated the benefits of adding LH to FSH stimulus in women with normal response to COS, higher levels of estradiol 14 15 16 17 18 and progesterone, 15 higher rate of high-quality embryos, 15 a smaller number of cycles cancelled, 14 increased pregnancy rate, 14 and less incidence of OHSS 14 were observed compared with stimulus with rFSH alone. One study demonstrated a negative impact of LH supplementation on oocyte maturation and fertilization. 13 Conversely, several studies reported no difference in the outcomes of cycles when rFSH alone was compared with rFSH + rLH. 8 19 20 21 22

In patients with poor response to COS, stimulation with rFSH + rLH resulted in higher pregnancy, implantation, and live birth rates when compared with stimulation with rFSH alone or human menopausal gonadotropin. 23 Another study showed that stimulation with rFSH + rLH yielded higher rate of high-quality embryos. 19 These results suggest that poor response to COS could be related to LH insufficiency, and rLH supplementation might rescue oocyte competence that, in turn, could lead to the development of viable embryos, thus increasing pregnancy outcomes. On the other hand, some studies reported no significant differences in ICSI outcomes when comparing the two stimulation regimens in poor responder patients. 24 25

Significantly increased implantation ratse 6 8 9 and live birth rates 9 have been observed in older women stimulated with rFSH + rLH when compared with their nonsupplemented counterparts. 8 Moreover, treatment with rLH significantly reduced total FSH consumption, 8 confirming that FSH and LH act synergistically. Conversely, Fábregues et al. 26 showed that rLH supplementation did not increase ovarian response to COS and implantation rates in patients of older reproductive ages. Marrs et al. 21 observed similar pregnancy rates in young and older women receiving rFSH + rLH; however, pregnancy rates in women ≥ 35 years old receiving rFSH alone significantly declined when compared with those of women < 35 years old, suggesting that these patients might benefit from the addition of rLH. It has been suggested that younger women possess a higher number of LH receptors compared with older women and, therefore, do not require LH supplementation, while LH supplementation in older women secures a sufficient LH-induced response. 8 Moreover, ovarian androgen secretion is also diminished in older women, suggesting an age-related decline in ovarian response to stimulation with LH. 8

Few studies investigated the potential benefits of adding rLH to rFSH in patients with reduced serum LH concentrations. Lisi et al. 27 showed an increased implantation rate in women with LH concentration < 1.0 IU/l at downregulation who received rLH supplementation, suggesting that, in the experience of profound LH downregulation, rLH supplementation might be beneficial. In opposition, Humaidan et al. 8 observed increased implantation rates when LH supplementation was used in patients with endogenous LH concentrations ≥ 1.99 IU/l. One study failed to demonstrate association between rLH supplementation and improved outcomes. 28

The lack of consensus in the aforementioned literature has led to the publication of several meta-analyses, which also came to conflicting conclusions. The meta-analyses suggested that rFSH+rLH results in shorter stimulation length and fewer rFSH consumption, 29 and yields higher estradiol levels 29 30 and higher number of mature oocytes. 30 While several meta-analysis showed no significant differences in implantation, 2 29 30 pregnancy, 2 29 30 and live-birth rates, 2 11 30 31 others have demonstrated higher implantation rates, 3 pregnancy rates, 3 32 ongoing pregnancy rates, 11 and lower miscarriage rates 11 in the recombinant LH-supplemented regimen.

For poor responder patients, an increase in clinical pregnancy rate was observed in favor of supplementing rLH. 2 32 In addition, poor responders showed significantly more retrieved oocytes with rFSH + rLH compared with rFSH alone. 32

The disparity found in the literature may be due to (i) LH administration start (beginning of treatment or late phase), (ii) type of GnRH analogue used (agonist or antagonist), (iii) starting dose of gonadotropin and gonadotropin dose adjustment during COS, (iv) heterogeneous definition of poor ovarian response, and (v) heterogeneous cutoff values for advanced maternal age (35 or 36 years old).

The possible mechanisms behind the benefits offered by rLH supplementation are improved oocyte competence and endometrial receptivity. Lower levels of cumulous cell apoptosis have been demonstrated in cycles with rLH supplementation as compared with cycles with rFSH alone, 33 which can reflect enhanced oocyte competence. In addition, LH stimulates CYP17 to convert progesterone into androgens, which in turn can be aromatized to estrogens. The supplementation with LH decreases the chance of a premature progesterone rise prior to luteinization, thus benefitting the endometrium and increasing the chance of implantation and clinical pregnancy. 34 Finally, the addition of rLH may improve follicular insulin sensitivity, leading to decreased androgen levels through a cascade mediated by increased production of adiponectin. This favorable setting may culminate in enhanced follicular maturation, ovulation, and fertilization capacity. 35

This is a retrospective study with its inherent limitations and bias. In addition, although the sample size was adequate for the analysis of the general group, the present study is underpowered for subgroups analyses. The present study was limited by its small sample size but creates a rationale to conduct randomized studies with larger casuistic to draw concrete conclusions about the use of rFSH and rLH for ovarian stimulation in patients with cycles stimulated with rFSH alone. The results presented here might provide another tool for the clinician to use in the decision-making process regarding the trigger regimen. The most important take home message is that the outcomes of ICSI cycles from unselected patients can be improved in a following cycle with the use of LH supplementation for ovarian stimulation.

## Conclusion

In conclusion, ovarian stimulation with LH supplementation results in higher implantation rates, regardless of maternal age and response to COS, compared with cycles stimulated with rFSH only. Improvements were also observed for ICSI laboratory outcomes and miscarriage rate when the patients were stratified by age and number of retrieved oocytes. Despite being encouraging, due to the retrospective nature of the present study, these results should be confirmed in randomized controlled trials.
